# Genomic medicine: educational challenges

**DOI:** 10.1002/mgg3.38

**Published:** 2013-09-10

**Authors:** Bruce R Korf

**Affiliations:** Department of Genetics, University of Alabama at BirminghamBirmingham, Alabama, 35294

I will begin and end this commentary on an optimistic, and perhaps heretical note: I am not concerned about physicians' lack of readiness to embrace genomic medicine. I believe that they will rise to the opportunity and that genomics will take its place as one of the major drivers of progress in modern medicine. I believe this because they have done it before – sweeping technological advancement is not new in medicine, and being prepared to embrace such change is fundamental to our medical education system.

I will use as an imperfect example the application of imaging – especially magnetic resonance imaging (MRI) – in routine medical practice. I trained in neurology, as well as in genetics, just as computerized imaging was being introduced in medicine. I saw my first MRI images as a resident and was amazed at their anatomical clarity. I do not understand the physics of MRI. I have some idea that atomic nuclei, magnetism, and radio waves are involved. I remember learning about nuclear magnetic resonance in college chemistry, but have a hard time relating the ppm graphs to the body or brain images I am now accustomed to seeing. I order a lot of MRIs – I think judiciously, but often – given the patients I tend to see (mostly those with neurofibromatosis or tuberous sclerosis complex). I am comfortable looking at the images, but also rely on radiologists for interpretation, as I am aware of my limited interpretive skills and of the pitfalls in the technology. I also appreciate the ability to call up reports and images in the clinic room, often walking my patients through the results.

I recognize the limits of this example in illuminating the clinical application of genomics. MRIs look a lot like the body parts I learned about in anatomy, whereas I was not taught in medical school to relate a list of genetic variants to risk of disease. Yet I think the example does serve well to illustrate four principles that do apply to genomic medicine: (1) the value of a basic science background but the ability to use a new technology without deep understanding of the underlying science; (2) the ability to gain competency in the use of a new technology in the context of clinical practice; (3) the need to recognize one's limitations and rely on experienced colleagues; (4) the value of point of care information systems.

## Basic Science and Genomic Medicine

It has been more than a century since Abraham Flexner issued a report that advanced American medical education from trade school status to professional training grounded in science. The medical education system has remained true to these values, but has been strained by a volume of scientific knowledge and technological advancement that would have been impossible to conceptualize in Flexner's time. In 2009 the Howard Hughes Medical Institute and the Association of American Medical Colleges issued a report “Scientific Foundation for Future Physicians (Howard Hughes Medical Institute/Association of American Medical Colleges 2009),” intended to highlight the basic science competences that should be expected for students matriculating into or graduating from medical school. The driving force was a perception expressed by some basic science faculty that science education in medical school was being diluted by pressure to make basic science clinically relevant. I was involved in drafting the competences, and as the one geneticist on the committee can be blamed for the any deficiencies in genetics and genomics in the document. One obvious gap is the lack of mention of genome sequencing, which began to permeate clinical medicine within a year of when the document was released. That said, all disciplines received equally brief, high level mention, by intention, as the competences were intended to spark curricular innovation by medical school faculty.

I think that the days are past when some medical schools taught little or no genetics in their basic science curriculum. At least this is not the major concern raised at the annual meeting of the Association of Professors of Human and Medical Genetics, where issues of medical education are discussed. I think it would be hard for any medical school curriculum committee to ignore the importance of genetics and genomics these days. If there is a deficiency it is more likely that concepts are taught with no mention of their clinical relevance. No basic scientific discipline lends itself better to melding of fundamental principles and clinical applications than genetics, and few have a clinical workforce that can so easily bridge the gap from the laboratory to the clinic.

What about physicians currently in practice, none of whom were taught about genomics in medical school, whether they were taught about or remember genetics? Here is an opportunity for targeted and pragmatic CME offerings, awakening memories of the role of DNA, genetic variation, and inheritance. Understanding of the technology of DNA sequencing can remain as rudimentary as my knowledge of the physics of MRI.

## Genomic Competency

Competency may be defined as the ability to achieve a desired outcome. We do not want our physicians to be merely competent, but with regard to a new technology like genomic sequencing, we do hope that they will add this competency to their overall technical and humanistic skill set. A competency-based approach to medical education has been spurred by the identification of six areas of core competency by the American Council of Graduate Medical Education (ACGME) (Patient Care, Medical Knowledge, Practice Based Learning and Improvement, Systems Based Practice, Professionalism, Interpersonal Skills and Communication) which inform all areas of residency education accredited by ACGME. Recently the AAMC proposed addition of two additional competences (Interprofessional Collaboration and Personal Development) (Englander et al. 2013).

Individual competencies may be so specific that they can be hard to relate to actual medical practice. For example, a general surgeon may be expected to be able to safely remove an inflamed appendix, and doing so requires many specific competencies, such as creating a sterile field, exposing the appendix, closing the wound, etc. What matters most, and may be observed during training, is the entirety of the surgical procedure, which in turn is comprised of many competencies that fit into specific ACGME core domains. A complex procedure such as appendectomy, has been referred to as a “entrustable medical activity,” that is, an activity that a physician in training has mastered and can now perform independently (ten Cate and Scheele 2007).

What are the entrustable medical activities (EPAs) that comprise practice of genomic medicine for the generalist physician? A working group of the Inter-Society Coordinating Committee, organized by the National Human Genome Research Institute to address medical education challenges across medical specialties, has developed a tentative list of such EPAs in genomics in five domains: family history, genomic testing, genomic-guided therapeutics, somatic genomics (cancer), and nonhuman genomics (microbiome). The working group is now developing competencies within each of these EPAs that map to the eight core competencies of ACGME and AAMC. Once completed, specialty societies will be invited to do the same with specialty-specific genomic competencies. It is hoped that this will result in a structure to guide future efforts to train physicians in genomic medicine, whether as residents or in continuing medical education.

## Developing a Genomic Medicine Workforce

Unlike some other areas of technology-driven change in medicine such as imaging or robotic surgery, genomic medicine does not require enormous capital expenditures to be available to physicians. The cost of DNA sequencing is plummeting, and it is easy to send a blood or saliva sample to an external laboratory for testing. The day may well come when any hospital lab may have an inexpensive desktop genome sequencer, with built in informatics tools to aid interpretation. Already, consumers can have wide-scale genotyping done at low cost, and even genome sequencing. I point all this out to emphasize that any physician can order genomic testing, even today, with or without deep knowledge of the implications of testing for his or her patient.

Perhaps in the future reporting tools will be so clear and comprehensive that any physician, or maybe even most consumers, will be able to use the information obtained from genome sequencing wisely. We are a long way from that point now. It is common to encounter variants of uncertain clinical significance that, if misinterpreted, can result in a patient being erroneously labeled as being at risk for a medical condition. There are also issues of privacy and confidentiality of genomic information, handling of incidental findings, testing of children, and many other areas of complexity that are likely to remain beyond the general physician's skill set. The sheer volume of genomic information will also exceed the capacity to review information in a standard brief clinical encounter.

Therefore, for all but the most straightforward tests (e.g., some pharmacogenetic tests), it is likely that the physician will defer to trained professionals who can interpret sequence data and review the implications with their patient. There is a cadre of medical geneticists who can meet this challenge. The American Board of Medical Genetics certifies physician medical geneticists as well as laboratory geneticists. Physicians first complete 2 years of training in an ACGME-accredited residency (such as pediatrics or internal medicine) and then a 2-year residency in medical genetics. Combined programs in pediatrics/medical genetics, internal medicine/medical genetics, and maternal fetal medicine/medical genetics also exist. Laboratory geneticists have a Ph.D. or M.D. degree and complete a 2-year fellowship, in clinical molecular genetics, biochemical genetics, or cytogenetics. There is also a 1-year fellowship in molecular genetic pathology, generally taken by individuals who have completed previous training in pathology. In addition, the American Board of Genetic Counseling certifies genetic counselors, whose training consists of a 2 years master's degree.

It has long been a concern that too few people are choosing to be trained in these areas, especially physicians in medical genetics (Korf et al. 2005). In part this reflects the low reimbursement by third party payers for many genetic services, a major problem given the debt that many medical students accumulate. It may also reflect a perception that geneticists deal only with rare disorders and rarely offer treatment. The opportunities to treat rare genetic conditions are rapidly increasing, however, and genomic medicine now deals with the entire genome, including both rare and common conditions.

The expansion of genomic medicine will require a trained specialty workforce, as well as new paradigms for counseling on a genome's worth of information, and a change in patterns of reimbursement. Genomic medicine should be perceived as preventative medicine, with modest upfront costs that can save both lives and dollars downstream. The American College of Medical Genetics and Genomics has defined competencies for the physician medical geneticist (Korf et al. 2011), including genomics competencies, and has developed CME offerings for its members who have completed their residency training to gain these competencies.

## Point of Care Information Systems

Genomics is the information system of the organism, so there is a natural convergence of medical genomics and medical information systems. What's more, the immense volume of genomic information exceeds human capacity to manage the data without help from automated systems. It is inconceivable that genomic information will be deployed in the clinic without the involvement of informatics tools to facilitate interpretation.

Genomic information will place unusual stresses on electronic health record systems. First, there is the large amount of information that would need to be stored. This is a problem that has already been solved by imaging systems, and storage capacity is increasing almost as quickly as DNA sequencing capacity. Second, genomic information will need to be available regardless of where a patient is seen. Current electronic health record systems tend to be institution-specific, and this already creates problems such as wasteful repetition of tests when a patient is seen at more than one facility. There is no reason to repeat a genomic test, except for cancer, yet this will happen if a system is not developed to permit sharing of genomic information across health systems. This could be done by granting access across institutions, but also could create opportunities for third parties that will store information and provide access on request from a patient.

Medicine in general is becoming too complex for a physician to practice without computational assistance, just as the pilot of a modern jumbo jet requires computer assistance to fly the plane. Genomics will significantly increase this need. Some genomic data will likely be embedded into electronic health systems in a way that is invisible to the physician. This will probably happen with pharmacogenetic data, where an electronic prescribing system will automatically calculate optimal drug dosage based on variables such as age, body weight, and genotype. Interpretation of genomic data to elucidate risk of disease or to clarify differential diagnosis of a patient with symptoms will require more visible interactive decision support systems. I think that these systems, more than educational needs, will prove to be rate limiting in the integration of genomics into medicine. When will physicians routinely use genomics in their day-to-day practice? When a simple, attractive, and even fun to use iTunes-like system is developed to guide physicians through the data maze.

## Conclusion

**Figure 1:**
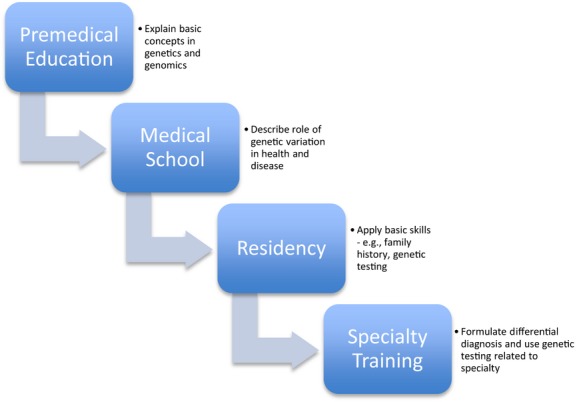
Progression of competency in genetics and genomics from premedical education through specialty training.

As promised, I will end as I began, with a note of optimism. As the power of genomic medicine becomes increasingly apparent it will sell itself to physicians, patients, and payers. We do need to educate the physician community both in the basic science of genomics and in its clinical application. There is a lot to be done, creating a progression of competency from premedical education through specialty training (Fig. 1), and there are many new educational modalities available to assist in the task. This is a golden age for innovators in medical education. Medical education has met such challenges in the past and it will here, too.

## References

[b1] ten Cate O, Scheele F (2007). Viewpoint: competency-based postgraduate training: can we bridge the gap between theory and clinical practice?. Acad. Med.

[b2] Englander R, Cameron T, Ballard AJ, Dodge J, Bull J, Aschenbrener CA (2013). Toward a common taxonomy of competency domains for the health professions and competencies for physicians. Acad. Med.

[b3] Howard Hughes Medical Institute/Association of American Medical Colleges (2009). Scientific foundations for future physicians.

[b4] Korf BR, Feldman G, Wiesner GL (2005). Report of Banbury Summit meeting on training of physicians in medical genetics, October 20–22, 2004. Genet. Med.

[b5] Korf BR, Irons M, Watson MS (2011). Competencies for the physician medical geneticist in the 21st century. Genet. Med.

